# Gender Differences in Non-Persistence with Angiotensin-Converting Enzyme Inhibitors and Angiotensin Receptor Blockers among Older Hypertensive Patients with Peripheral Arterial Disease

**DOI:** 10.3390/biomedicines10071479

**Published:** 2022-06-22

**Authors:** Martin Wawruch, Jan Murin, Tomas Tesar, Martina Paduchova, Miriam Petrova, Denisa Celovska, Beata Havelkova, Michal Trnka, Lucia Masarykova, Sofa D. Alfian, Emma Aarnio

**Affiliations:** 1 Institute of Pharmacology and Clinical Pharmacology, Faculty of Medicine, Comenius University, 811 08 Bratislava, Slovakia; miriampetrova1@gmail.com; 2 1st Department of Internal Medicine, Faculty of Medicine, Comenius University, 813 69 Bratislava, Slovakia; jan.murin@gmail.com (J.M.); denisa.celovska@gmail.com (D.C.); 3 Department of Organisation and Management of Pharmacy, Faculty of Pharmacy, Comenius University, 832 32 Bratislava, Slovakia; tesar@fpharm.uniba.sk; 4 Department of Angiology, Health Centre, 917 01 Trnava, Slovakia; martina.paduchova@medena.sk; 5 General Health Insurance Company, 851 04 Bratislava, Slovakia; beata.havelkova@vszp.sk; 6 Institute of Medical Physics, Biophysics, Informatics and Telemedicine, Faculty of Medicine, Comenius University, 813 72 Bratislava, Slovakia; michal.trnka@fmed.uniba.sk; 7 Department of Pharmacology and Clinical Pharmacy, Faculty of Pharmacy, Universitas Padjadjaran, Jatinangor 45363, Indonesia; sofa.alfian@unpad.ac.id; 8 Center of Excellence in Higher Education for Pharmaceutical Care Innovation, Universitas Padjadjaran, Jatinangor 45363, Indonesia; 9 School of Pharmacy, University of Eastern Finland, 70211 Kuopio, Finland; emma.aarnio@uef.fi

**Keywords:** peripheral arterial disease, adherence, persistence, angiotensin-converting enzyme inhibitors, angiotensin receptor blockers, general practitioner, new user, arterial hypertension, older patient

## Abstract

The beneficial effects of angiotensin-converting enzyme inhibitors (ACEIs) and angiotensin receptor blockers (ARBs) in hypertensive patients with peripheral arterial disease (PAD) depends on long-term persistence. The aims of our study were to analyse gender differences in non-persistence with ACEIs/ARBs, and to identify the characteristics associated with the likelihood of non-persistence. Our study cohort included 7080 hypertensive patients (4005 women and 3075 men) aged ≥65 years, treated with ACEIs/ARBs, in whom PAD was diagnosed between 1 January and 31 December 2012. Non-persistence was identified according to a treatment gap of 6 months without ACEI/ARB prescriptions. The characteristics associated with non-persistence were identified using the Cox regression model. At the end of the 5-year follow-up, 23.2% of the whole study cohort, 22.3% of men, and 23.9% of women were non-persistent with ACEIs/ARBs, with no significant gender differences in persistence. While a number of characteristics were associated with non-persistence, only three characteristics had consistent, statistically significant associations in both genders: being a new ACEI/ARB user increased the likelihood of non-persistence, and general practitioner as index prescriber and increasing the overall number of medications decreased the likelihood of non-persistence. Information on the differences in characteristics that are associated with non-persistence between genders may help to better identify patients for whom special attention should be paid to improve their persistence.

## 1. Introduction

In this article, peripheral arterial disease (PAD) refers to the atherosclerotic disease of the arteries of lower limbs. It is estimated that PAD affects 10–15% of the general population. Around 200 million people worldwide suffer from PAD. The prevalence of PAD increases with advancing age and it is estimated that 20% of people aged above 80 years are affected by PAD. PAD is associated with an annual mortality rate of 4–6% [1,2,3,4,5]. PAD patients are at increased risk for major adverse cardiac events (MACE), including myocardial infarction (MI), ischaemic stroke, and cardiovascular (CV) death, and also at increased risk for major adverse limb events (major amputations and acute limb ischaemia). In the case of patients with symptomatic PAD, annual rates of MACE and major adverse limb events are 4–5% and 1–2%, respectively [6,7].

The risk factors of PAD include smoking, arterial hypertension, hypercholesterolemia, diabetes mellitus, and chronic kidney disease. The treatment of PAD includes lifestyle modifications (smoking cessation and dietary changes) and the administration of antiplatelet agents, statins, and antihypertensive medications [6,8,9]. According to the Guidelines of the European Society of Cardiology, in hypertensive PAD patients, blood pressure control at <140/90 mmHg is recommended (IA). Diuretics, beta-blockers, calcium antagonists, angiotensin-converting enzyme inhibitors (ACEIs), and angiotensin receptor blockers (ARBs) are all suitable in the treatment of hypertension in these patients. However, ACEIs and ARBs should be considered as first line therapies in patients with PAD and arterial hypertension (IIaB). Some antihypertensive agents (e.g., the calcium channel blocker verapamil), prostaglandins (I2 and E1), and cilostazol claim to increase the walking distance [8].

The beneficial effect of the pharmacologic treatment of PAD depends on adequate patient adherence to the medications prescribed by the physician. Adherence includes three phases: initiation, implementation, and persistence. Persistence characterises the length of time between initiation and the last dose that precedes discontinuation. The period of non-persistence starts after discontinuation [10,11]. Long-term persistence with antihypertensive medications in PAD patients is of particular importance, since this treatment requires the life-long administration of drugs [12].

To the best of our knowledge, in the literature, there are no studies focused on the analysis of non-persistence and the factors associated with non-persistence with ACEIs/ARBs, specifically in older PAD patients. Several studies analysing gender differences in non-persistence with antihypertensive medications have been published. However, the results of those studies are inconsistent. In some studies, females were more likely to be persistent with antihypertensive treatment [13,14], but in the study by Erkens et al. [15], persistence was lower in women than in men. Those studies evaluated persistence with antihypertensive drugs, but not specifically in PAD patients. We considered it interesting to analyse whether there are differences in the factors associated with non-persistence between the two genders. For these reasons, the aims of our study were: (a) to analyse gender differences in non-persistence with ACEIs/ARBs in older PAD patients with arterial hypertension, and (b) to identify patient- and medication-related characteristics associated with the likelihood of non-persistence in the whole study cohort and separately in the groups of men and women.

## 2. Materials and Methods

### 2.1. Database and Study Population

**Figure 1:**
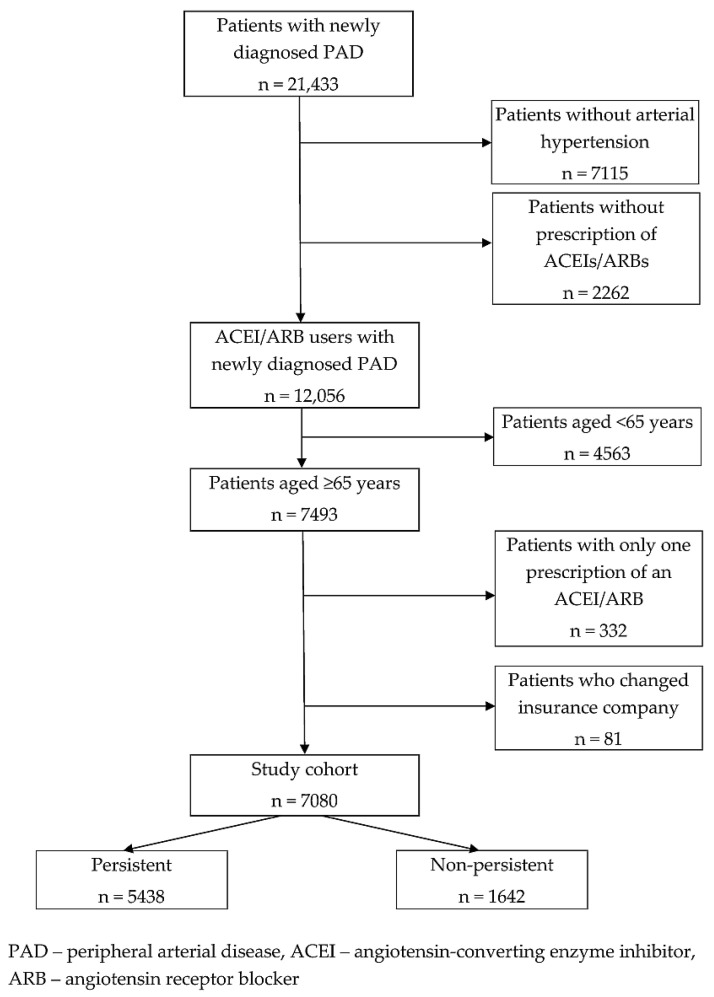
Flow chart showing derivation of the study cohort (*n* = 7080).

Our study cohort of 7080 patients was drawn from a sample of 21,433 patients in whom PAD was diagnosed between 1 January and 31 December 2012. The sample included 12,056 patients with arterial hypertension treated with ACEIs or ARBs. From among them, patients aged ≥65 years were selected (*n* = 7493). After excluding patients with only one prescription of an ACEI/ARB (*n* = 332) and those who changed their insurance company during the follow-up period (*n* = 81), the remaining sample of 7080 patients represented the study cohort for our evaluations (Figure 1). The data for our study were collected from the database of the General Health Insurance Company, the largest health insurance provider in Slovakia, which covers approximately 63% of the population.

### 2.2. Analysis of Non-Persistence

In our retrospective cohort study, non-persistence was identified based on the presence of a treatment gap period of 6 months without a prescription of ACEIs/ARBs, observed after the estimated date of the last day covered by the last package of the prescribed medication. All tablets in each package of prescribed medication were considered in our analyses, focusing on the evaluation of the length of the period covered by the medication. Patients in whom such treatment gap was recorded were classified as being non-persistent, and those without such a gap were considered to be persistent. An analysis of non-persistence was performed in the whole study cohort, and separately in the groups of men and women.

The index date of the study was the date of the first ACEI/ARB prescription after the diagnosis of PAD. From the index date, patients were followed for 5 years, or up to the date of their death if it occurred during the follow-up period. Patients who died during the follow-up were censored in order to avoid misclassifying them as non-persistent.

### 2.3. Factors Associated with Non-Persistence

The following patient- and medication-related characteristics were analysed as being potentially associated with non-persistence:

- Socio-demographic characteristics: age, gender, university education, and employment.
- History of CV events: ischaemic stroke, transient ischaemic attack (TIA) and MI.
- Comorbid conditions and their number. Data on comorbid conditions were collected according to the 10th revision of the International Classification of Diseases [16].
- Medication-related characteristics: initially administered ACEI/ARB agent, whether the patient was a new user of ACEIs/ARBs (the administration of ACEIs/ARBs was initiated in association with the diagnosis of PAD, with no prescription of ACEIs/ARBs being recorded during the two years before the PAD diagnosis) or a prevalent user (the administration of ACEIs/ARBs was initiated before the PAD diagnosis), the patient’s co-payment, and the specialisation of the initial prescriber (a general practitioner or a specialist).
- CV co-medication: overall number of medications, number of CV medications, and particular CV medication classes recorded according to their ATC codes [17].

The data on these characteristics were collected at the time of inclusion in the study.

### 2.4. Statistical Analysis

Continuous variables were expressed as means ± standard deviations (SD). Categorical variables were characterised as frequencies and percentages.

The differences in the categorical variables between the two groups (persistent and non-persistent patients) were analysed using the χ^2^-test. To compare continuous variables between the two groups, the Mann–Whitney U test was applied. The reason for the use of this non-parametric test was the non-Gaussian distribution of analysed continuous variables. To analyse the normality of the distribution of continuous variables, the Kolmogorov–Smirnov test was used.

The number of patients who discontinued treatment with ACEIs/ARBs during the particular years of the follow-up period was determined using the Life Table analysis. To compare the development of the probability of persistence during the follow-up period in men and women, as well as to compare the likelihood of persistence between ACEI and ARB users (in the whole study group and in both gender groups), the Kaplan–Meier model was applied. In the Kaplan–Meier model, the statistical significance of the difference in the probability of persistence between the two groups was evaluated using the log-rank test. To identify the most important characteristics associated with non-persistence, the Cox proportional hazards model was applied. This model included all patient- and medication-related characteristics evaluated as factors that were potentially associated with non-persistence. The hazard ratios (HR) and corresponding 95% confidence intervals were determined for each characteristic [18]. The Cox regression model was performed in the whole study cohort, and separately in both gender groups.

All statistical tests were performed at the level of statistical significance: α = 0.05, which represents a cut-off value standardly used in biomedical research. The statistical software IBM SPSS for Windows, version 28 (IBM SPSS Inc., Armonk, NY, USA) was used.

### 2.5. Sensitivity Analyses

The effect of shorter and longer lengths of the treatment gap period (1–5 and 12 months) defining non-persistence on the proportions of non-persistent patients was assessed in the whole study cohort and separately in both gender groups. In our study, a relatively long follow-up period of 5 years was used. To analyse the possible influence of a shorter follow-up period on the results of our study, a Cox regression model with a 3-year follow-up period was performed in the whole study cohort and separately in the groups of men and women.

## 3. Results

In our study sample of 7080 patients, women (*n* = 4005; 56.6%) prevailed over men (*n* = 3075; 43.4%) and women were older than men (75.9 ± 6.9 vs. 74.3 ± 6.5; *p* < 0.001, according to the Mann–Whitney U test). The baseline characteristics of the study cohort are shown in Table 1.

During the first, second, third, fourth, and fifth year of the follow-up period, those identified as non-persistent with ACEIs/ARBs in the whole study cohort were 9.4%, 5.2%, 3.8%, 3.1% and 1.7% of patients, respectively; in the group of men, 8.9%, 4.6%, 3.9%, 3.2% and 1.7% of patients, respectively; and in the group of women, 9.7%, 5.5%, 3.8%, 3.1% and 1.8% of patients, respectively. At the end of the follow-up period, 1642 patients (23.2%) were non-persistent with ACEI/ARB treatment in the whole study cohort (*n* = 7080). Out of 3075 men, 685 patients (22.3%), and out of 4005 women, 957 patients (23.9%) became non-persistent during the follow-up period.

**Figure 2:**
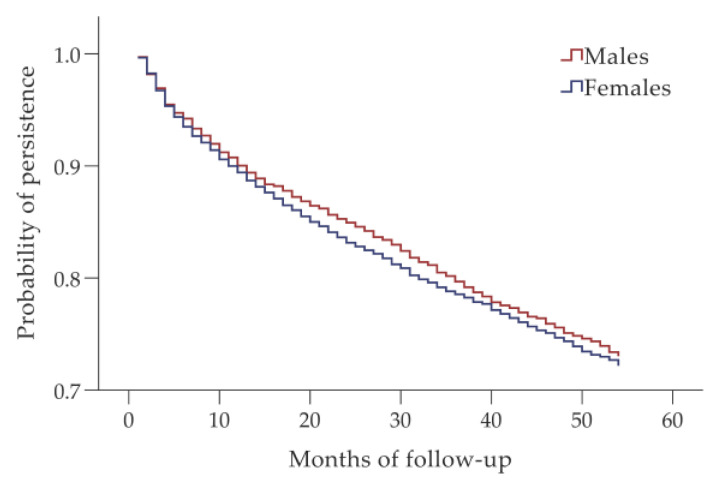
Persistence with angiotensin-converting enzyme inhibitors/angiotensin receptor blockers in males and females.

**Figure 3:**
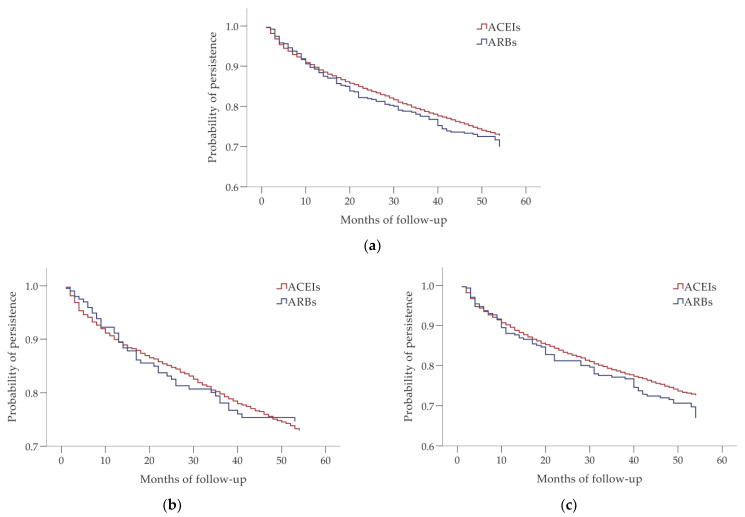
Differences in persistence between users of angiotensin-converting enzyme inhibitors (ACEIs) and angiotensin receptor blockers (ARBs) (**a**) in the whole study cohort; (**b**) among males; and (**c**) among females.

According to the Kaplan–Meier analysis, there was no significant difference in the development of the probability of persistence during the follow-up period between men and women (*p* = 0.940 according to the log-rank test) (Figure 2). No significant differences in persistence were found between ACEI and ARB users in the whole study cohort, and in the groups of men and women (*p* = 0.264, *p* = 0.767 and *p* = 0.099, respectively, according to the log-rank test) (Figure 3).

In the Cox regression model, the following characteristics were associated with persistence: (a) in the whole study cohort: atrial fibrillation, diabetes mellitus, dementia, administration of enalapril, general practitioner as index prescriber, an increasing overall number of medications, the administration of beta-blockers, thiazide diuretics, and calcium channel blockers; (b) in the group of men: administration of ramipril, trandolapril, quinapril, and beta-blockers, general practitioner as index prescriber and an increasing overall number of medications; and (c) in the group of women: increasing age, atrial fibrillation, diabetes mellitus, dementia, general practitioner as index prescriber, an increasing overall number of medications, the administration of thiazide diuretics and calcium channel blockers. On the other hand, the following characteristics were associated with an increased probability of non-persistence: (a) in the whole study cohort: the administration of imidapril, fosinopril, or valsartan, being a new user of ACEIs/ARBs, an increasing number of CV medications, and the administration of statins; (b) in the group of men: the administration of imidapril and being a new user of ACEIs/ARBs; and (c) in the group of women: the administration of valsartan and statins, being a new user of ACEIs/ARBs and an increasing number of CV medications (Table 2).

### Sensitivity Analyses

An inverse relationship between the length of the treatment gap period and the proportion of non-persistent patients was confirmed in the sensitivity analysis using different lengths of the treatment gap period (1–6 and 12 months) (Supplementary Table S1). The use of treatment gap periods shorter than 6 months led to the overestimation, and of a 12-month treatment gap period, to the underestimation of non-persistence. For this reason, the choice of the 6-month treatment gap period may be considered as being appropriate for defining non-persistence in our study.

In the Cox regression model with a shorter 3-year follow-up period, mainly similar characteristics associated with non-persistence as those found in the main model with a 5-year follow-up period were identified (Supplementary Table S2). Most of the differences between the 5-year and 3-year models were caused by the characteristics being associated with the probability of non-persistence using the 5-year follow-up, but not during the 3-year follow-up. However, the administration of cardiac glycosides in the whole study cohort was associated with persistence in the model with a 3-year follow-up period, but not in the main model. Among men and women, some characteristics lost their association with non-persistence when using the 3-year follow-up instead of the 5-year follow-up. In addition, chronic heart failure, diabetes mellitus, the administration of valsartan, thiazide diuretics, or calcium channel blockers, and the number of CV medications were associated with the likelihood of non-persistence in the 3-year model, but not in the 5-year model, among men. Among women, the administration of imidapril was associated with the likelihood of non-persistence in the 3-year model, but not in the 5-year model.

## 4. Discussion

In our study, more than one-fifth of patients became non-persistent during the 5-year follow-up period over the whole study cohort, as well as in the groups of men and women (23.2%, 22.3%, and 23.9%, respectively). We did not find any significant difference in the development of persistence during the follow-up period between the genders, nor between ACEI and ARB users. However, differences were found in factors associated with the probability of non-persistence among the whole study cohort and among the groups of men and women. Being a new user of ACEIs/ARBs, general practitioner as index prescriber and an increasing overall number of medications represented the only factors that were consistently associated with the likelihood of non-persistence in all of the three evaluated groups (the whole study cohort and the groups of men and women).

Among the socio-demographic characteristics, increasing age represented a factor associated with persistence only in the group of women. This result may indicate careful medication-taking behaviour in older women. Greater age increased the chance of being persistent with ACEIs and ARBs in the study by Vegter et al. [19], which analysed drug-utilisation patterns of ACEIs and ARBs among new Dutch users. Very old patients aged ≥80 years were at high risk of discontinuation, but patients aged 65–79 years had a higher likelihood of persistence with antihypertensive treatment in a retrospective cohort study by Ah et al. [13]. In contrast to our study, patients older than 75 years represented a subgroup that demonstrated poorer persistence in the population-based cohort study by Tu et al. [20]. That study included new users of antihypertensive medication aged ≥66 years. In the study by Qvarnström et al. [21], the highest persistence was observed in patients aged 60–69 years (more than 70% continued at two years of the follow-up period), whereas the lowest persistence was reported in patients aged 30–49 years (less than 50% continued at two years of the follow-up period). Their cohort study analysed the factors associated with low persistence in patients initiated on antihypertensive drugs in Swedish primary healthcare.

No significant gender differences in persistence with ACEIs/ARBs were found in our study cohort. However, females were more likely to be persistent with antihypertensive treatment, in the study by Ah et al. [13]. In addition, Qvarnström et al. [14] reported the discontinuation of antihypertensive drug classes as being more common in men than in women. According to the authors of that observational cohort study, this finding supports the idea that has been previously proposed, that men are less involved in their preventive care in comparison with women [22]. Opposing results have also been published as, according to the study by Erkens et al. [15], persistence with antihypertensive drugs was lower in women than in men.

Among comorbid conditions, atrial fibrillation, diabetes mellitus, and dementia were associated with persistence in the whole cohort of our study, and in the group of women. Atrial fibrillation and diabetes mellitus represent conditions that require long-term pharmacologic treatment. Patients with these conditions are used to regularly taking medications, and their persistence with ACEIs/ARBs may therefore also be expected. Similar to our study, having diabetes mellitus increased the rate of persistence, in the study by Perreault et al. [23]. That study evaluated persistence with antihypertensive medications among newly treated patients with essential hypertension. Unlike our study, middle-aged patients between 50 and 64 years were included. Having diabetes mellitus significantly increased the rate of persistence in the primary prevention cohort, in the study by Gogovor et al. [24]. Their cohort study analysed persistence rates with ACEIs used in the primary and secondary prevention of CV diseases. In the case of dementia, patients’ caregivers took care of patients’ regular taking of medications. Similar to our results, in the study by Ah et al. [13], patients with dementia had a higher likelihood of persistence with antihypertensive treatment.

We did not find any significant difference in the development of persistence during the follow-up period between users of ACEIs and ARBs. On the other hand, Erkens et al. [15] reported the highest persistence in the case of ARB users, and a progressively lower persistence among users of ACEIs, beta-blockers, calcium channel blockers, and diuretics. Burke et al. [25] analysed the discontinuation of antihypertensive drugs among newly diagnosed hypertensive patients. They reported the longest median time to antihypertensive class discontinuation in the case of ARBs, followed by ACEIs, calcium channel blockers, beta-blockers, thiazides, alpha-antagonists, and other antihypertensive medications.

Several ACEIs and ARBs were associated with the likelihood of non-persistence. Enelapril in the whole study cohort, and ramipril, trandolapril, and quinapril in men were associated with persistence. On the contrary, fosinopril in the whole study cohort, imidapril in the whole study cohort and in the group of men, and valsartan in the whole study cohort and in the group of women were associated with non-persistence. The design of our study does not make it possible to explain why the administration of these medications was associated with a decreased or an increased probability of non-persistence. Vegter et al. [19] also found significant differences in persistence among ACEIs. Enalapril users had the lowest persistence after 3 years, while users of ramipril and fosinopril had the highest persistence. In the study by Ah et al. [26], valsartan initiators were less likely to discontinue the initial drug, compared with losartan initiators. That nation-wide population-based study evaluated the influence of initial ARB on the treatment persistence among patients with uncomplicated hypertension.

Being a new user of ACEIs/ARBs represented a factor that was associated with non-persistence in all three of the evaluated groups, i.e., the whole cohort of our study, and the groups of men and women. This result may be associated with the possible adverse effects of ACEIs/ARBs, which may occur at the beginning of treatment. These adverse effects may lead to discontinuation of the treatment. In the study by Qvarnström et al. [14], approximately 40% of all patients discontinued their initial antihypertensive drug class during the first year. Burke et al. [25] concluded that general practitioners should closely monitor patients during the first year following antihypertensive drug initiation, because of the high early risk of discontinuation.

In our study, general practitioner as index prescriber was associated with persistence consistently in all three evaluated groups. This result indicates the key role of general practitioners and their explanation of the importance of persistence with ACEIs/ARBs in older PAD patients. On the other hand, Van Wijk et al. [27] reported on patients who had been initially treated by a cardiologist or an internist having higher persistence with antihypertensive treatment compared with general practitioners. The authors of that retrospective cohort study analysed the rate and determinants of 10-year persistence with antihypertensive medications.

An increasing overall number of medications taken represented a factor associated with persistence in all three of the groups evaluated in our study. This result may indicate a meticulous medication-taking behaviour in patients with polypharmacy, who are used to concomitantly taking several medications. Surprisingly, an increasing number of CV medications was associated with non-persistence in the whole study cohort, and in the group of women. The design of our study does not make it possible to explain this discrepancy in the association between the overall number of medications and the number of CV medications with the likelihood of non-persistence. Subjects having a higher number of different classes of drugs/month (≥4) were less likely to discontinue antihypertensive therapy in both the primary and secondary prevention cohorts, in the study by Gogovor et al. [24].

Among CV co-medication, the following classes were associated with persistence: beta-blockers (the whole cohort of our study and the group of men), thiazide diuretics (the whole cohort of our study and the group of women), and calcium channel blockers (the whole cohort of our study and the group of women). On the contrary, the administration of statins was associated with non-persistence (the whole cohort of our study and the group of women). Co-medication used in the treatment of dyslipidaemia and the use of diuretics decreased the chance of being persistent with ACEIs/ARBs, in the study by Vegter et al. [19].

Our study has some limitations that should be taken into consideration when interpreting the results of the study. The database of the General Health Insurance Company, which represented the source of data for our study, was originally created for insurance and reimbursement purposes, and not for research. The design of our study does not make it possible to distinguish who was responsible for the discontinuation of ACEI/ARB treatment, i.e., the physician or the patient. It was also impossible to identify whether patients really took their medications as prescribed by the physician. The database of the General Health Insurance Company does not include information on the grade and severity of PAD, which may affect adherence to treatment. For this reason, we were unable to evaluate these characteristics. We had no access to data beyond the end of the study period. Consequently, it was impossible to identify the treatment gap during the period of less than 6 months before the end of the follow-up of our study. Pharmacological treatment in hypertensive PAD patients besides ACEIs/ARBs also include antiplatelet medication and statins [8,9]. However, the study presented in this manuscript was focused solely on ACEIs/ARBs. It was impossible to analyse the effects of the discontinuation of ACEIs/ARBs on patients’ clinical outcomes and prognosis, since the database of the General Health Insurance Company does not include all of the necessary information for such analysis (e.g., the values of blood pressure). On the other hand, the large sample size covering all regions of the Slovak Republic and precise data on patients’ comorbid conditions and medications represent the strengths of our study.

## 5. Conclusions

Approximately one-fifth of both the male and female patients in our study became non-persistent during the 5-year follow-up period. We did not find any significant differences in persistence between men and women, nor between ACEI and ARB users. However, there were differences in the patient- and medication-related characteristics associated with non-persistence among the whole study cohort and the groups of men and women. The only factors consistently associated with the probability of non-persistence in both genders were being a new user of ACEIs/ARBs increasing that probability, and general practitioner as index prescriber and an increasing overall number of medications decreasing that probability. The identification of differences in the characteristics associated with non-persistence between the genders can help with identifying patients to whom special attention should be paid to improve their persistence.

## Figures and Tables

**Table 1:** Baseline characteristics of the study cohort.

**Factor**	**The Whole Study Cohort**				**Males**				**Females**			
	**All** **(*n* = 7080)**	**Persistent (*n* = 5438)**	**Non-persistent (*n* = 1642)**	***p***	**All** **(*n* = 3075)**	**Persistent (*n* = 2390)**	**Non-persistent (*n* = 685)**	***p***	**All** **(*n* = 4005)**	**Persistent (*n* = 3048)**	**Non-persistent (*n* = 957)**	***p***
*Socio-demographic characteristics*												
Age	75.2 ± 6.8	75.5 ± 6.9	74.1 ± 6.2	<0.001 *	74.3 ± 6.5	74.6 ± 6.6	73.5 ± 6.1	<0.001 *	75.9 ± 6.9	76.3 ± 7.1	74.5 ± 6.2	<0.001 *
Female sex	4005 (56.6)	3048 (56.1)	957 (58.3)	0.110								
University education	490 (6.9)	373 (6.9)	117 (7.1)	0.709	380 (12.4)	291 (12.2)	89 (13.0)	0.567	110 (2.7)	82 (2.7)	28 (2.9)	0.697
Employed patients	347 (4.9)	264 (4.9)	83 (5.1)	0.742	235 (7.6)	182 (7.6)	53 (7.7)	0.916	112 (2.8)	82 (2.7)	30 (3.1)	0.467
*History of CV events* ^a^												
History of ischemic stroke	1238 (17.5)	992 (18.2)	246 (15.0)	**0.002**	569 (18.5)	467 (19.5)	102 (14.9)	**0.006**	669 (16.7)	525 (17.2)	144 (15.0)	0.115
History of TIA	462 (6.5)	353 (6.5)	109 (6.6)	0.833	172 (5.6)	132 (5.5)	40 (5.8)	0.751	290 (7.2)	221 (7.3)	69 (7.2)	0.966
History of MI	423 (6.0)	339 (6.2)	84 (5.1)	0.094	210 (6.8)	171 (7.2)	39 (5.7)	0.181	213 (5.3)	168 (5.5)	45 (4.7)	0.330
*Comorbid conditions*												
Number of comorbid conditions	2.7 ± 1.6	2.8 ± 1.6	2.6 ± 1.6	<0.001 *	2.6 ± 1.6	2.6 ± 1.5	2.3 ± 1.6	<0.001 *	2.9 ± 1.6	2.9 ± 1.6	2.8 ± 1.6	0.476 *
Chronic heart failure	585 (8.3)	473 (8.7)	112 (6.8)	**0.015**	248 (8.1)	206 (8.6)	42 (6.1)	**0.035**	337 (8.4)	267 (8.8)	70 (7.3)	0.160
Atrial fibrillation	1145 (16.2)	942 (17.3)	203 (12.4)	**<0.001**	545 (17.7)	450 (18.8)	95 (13.9)	**0.003**	600 (15.0)	492 (16.1)	108 (11.3)	**<0.001**
Diabetes mellitus	2879 (40.7)	2280 (41.9)	599 (36.5)	**<0.001**	1221 (39.7)	987 (41.3)	234 (34.2)	**<0.001**	1658 (41.4)	1293 (42.4)	365 (38.1)	**0.019**
Hypercholesterolemia	2589 (36.6)	1982 (36.4)	607 (37.0)	0.701	1050 (34.1)	837 (35.0)	213 (31.1)	0.056	1539 (38.4)	1145 (37.6)	394 (41.2)	**0.046**
Dementia	571 (8.1)	475 (8.7)	96 (5.8)	**<0.001**	202 (6.6)	170 (7.1)	32 (4.7)	**0.023**	369 (9.2)	305 (10.0)	64 (6.7)	**0.002**
Depression	802 (11.3)	605 (11.1)	197 (12.0)	0.328	199 (6.5)	155 (6.5)	44 (6.4)	0.954	603 (15.1)	450 (14.8)	153 (16.0)	0.356
Anxiety disorders	2122 (30.0)	1627 (29.9)	495 (30.1)	0.860	618 (20.1)	488 (20.4)	130 (19.0)	0.407	1504 (37.6)	1139 (37.4)	365 (38.1)	0.667
Parkinson’s disease	303 (4.3)	237 (4.4)	66 (4.0)	0.552	107 (3.5)	87 (3.6)	20 (2.9)	0.364	196 (4.9)	150 (4.9)	46 (4.8)	0.886
Epilepsy	195 (2.8)	150 (2.8)	45 (2.7)	0.969	99 (3.2)	78 (3.3)	21 (3.1)	0.796	96 (2.4)	72 (2.4)	24 (2.5)	0.797
Bronchial asthma/COPD	1425 (20.1)	1096 (20.2)	329 (20.0)	0.917	648 (21.1)	521 (21.8)	127 (18.5)	0.065	777 (19.4)	575 (18.9)	202 (21.1)	0.126
*ACEI/ARB related characteristics*												
Initially administered ACEI/ARB												
Perindopril	2963 (41.9)	2249 (41.4)	714 (43.5)	**<0.001**	1263 (41.1)	949 (39.7)	314 (45.8)	**0.006**	1700 (42.4)	1300 (42.7)	400 (41.8)	**0.032**
Lisinopril	367 (5.2)	269 (4.9)	98 (6.0)		143 (4.7)	103 (4.3)	40 (5.8)		224 (5.6)	166 (5.4)	58 (6.1)	
Ramipril	1184 (16.7)	927 (17.0)	257 (15.7)		586 (19.1)	468 (19.6)	118 (17.2)		598 (14.9)	459 (15.1)	139 (14.5)	
Enalapril	102 (1.4)	89 (1.6)	13 (0.8)		43 (1.4)	37 (1.5)	6 (0.9)		59 (1.5)	52 (1.7)	7 (0.7)	
Spirapril	11 (0.2)	8 (0.1)	3 (0.2)		5 (0.2)	4 (0.2)	1 (0.1)		6 (0.1)	4 (0.1)	2 (0.2)	
Trandolapril	1226 (17.3)	975 (17.9)	251 (15.3)		583 (19.0)	476 (19.9)	107 (15.6)		643 (16.1)	499 (16.4)	144 (15.0)	
Quinapril	569 (8.0)	448 (8.2)	121 (7.4)		196 (6.4)	161 (6.7)	35 (5.1)		373 (9.3)	287 (9.4)	86 (9.0)	
Imidapril	93 (1.3)	60 (1.1)	33 (2.0)		35 (1.1)	22 (0.9)	13 (1.9)		58 (1.4)	38 (1.2)	20 (2.1)	
Fosinopril	63 (0.9)	41 (0.8)	22 (1.3)		21 (0.7)	14 (0.6)	7 (1.0)		42 (1.0)	27 (0.9)	15 (1.6)	
Valsartan	159 (2.2)	105 (1.9)	54 (3.3)		68 (2.2)	48 (2.0)	20 (2.9)		91 (2.3)	57 (1.9)	34 (3.6)	
Losartan	137 (1.9)	113 (2.1)	24 (1.5)		59 (1.9)	51 (2.1)	8 (1.2)		78 (1.9)	62 (2.0)	16 (1.7)	
Telmisartan	107 (1.5)	80 (1.5)	27 (1.6)		41 (1.3)	32 (1.3)	9 (1.3)		66 (1.6)	48 (1.6)	18 (1.9)	
Candesartan	87 (1.2)	66 (1.2)	21 (1.3)		26 (0.8)	21 (0.9)	5 (0.7)		61 (1.5)	45 (1.5)	16 (1.7)	
Irbesartan	12 (0.2)	8 (0.1)	4 (0.2)		6 (0.2)	4 (0.2)	2 (0.3)		6 (0.1)	4 (0.1)	2 (0.2)	
New user of ACEIs/ARBs ^b^	456 (6.4)	265 (4.9)	191 (11.6)	**<0.001**	221 (7.2)	137 (5.7)	84 (12.3)	**<0.001**	235 (5.9)	128 (4.2)	107 (11.2)	**<0.001**
Patient’s co-payment (EUR) ^c^	3.0 ± 2.8	3.0 ± 2.7	2.9 ± 2.5	0.474 *	3.0 ± 2.6	3.0 ± 2.4	2.8 ± 1.8	0.626 *	3.0 ± 2.7	3.0 ± 2.6	2.9 ± 2.0	0.565 *
General practitioner as index prescriber	5821	4568	1253	**<0.001**	2519 (81.9)	2002 (83.8)	517 (75.5)	**<0.001**	3302 (82.4)	2566 (84.2)	736 (76.9)	**<0.001**
*CV co-medication*												
Number of medications	7.9 ± 2.7	7.9 ± 2.6	7.5 ± 2.9	<0.001 *	7.6 ± 2.8	7.8 ± 2.7	7.2 ± 3.1	<0.001 *	8.0 ± 2.6	8.1 ± 2.5	7.8 ± 2.8	0.036 *
Number of CV medications	4.8 ± 2.2	4.9 ± 2.3	4.6 ± 2.2	<0.001 *	4.7 ± 2.2	4.8 ± 2.2	4.4 ± 2.1	<0.001 *	4.9 ± 2.3	5.0 ± 2.3	4.8 ± 2.2	0.118 *
Antiplatelet agents	4989 (70.5)	3843 (70.7)	1146 (69.8)	0.495	2242 (72.9)	1744 (73.0)	498 (72.7)	0.889	2747 (68.6)	2099 (68.9)	648 (67.7)	0.503
Anticoagulants	1878 (26.5)	1494 (27.5)	384 (23.4)	**0.001**	866 (28.2)	697 (29.2)	169 (24.7)	**0.021**	1012 (25.3)	797 (26.1)	215 (22.5)	**0.022**
Cardiac glycosides	681 (9.6)	573 (10.5)	108 (6.6)	**<0.001**	263 (8.6)	228 (9.5)	35 (5.1)	**<0.001**	418 (10.4)	345 (11.3)	73 (7.6)	**0.001**
Antiarrhythmic agents	575 (8.1)	463 (8.5)	112 (6.8)	**0.028**	272 (8.8)	222 (9.3)	50 (7.3)	0.106	303 (7.6)	241 (7.9)	62 (6.5)	0.145
Beta-blockers	1392 (19.7)	1105 (20.3)	287 (17.5)	**0.011**	548 (17.8)	457 (19.1)	91 (13.3)	**<0.001**	844 (21.1)	648 (21.3)	196 (20.5)	0.606
Thiazide diuretics	1545 (21.8)	1237 (22.7)	308 (18.8)	**<0.001**	596 (19.4)	488 (20.4)	108 (15.8)	**0.007**	949 (23.7)	749 (24.6)	200 (20.9)	**0.020**
Loop diuretics	1662 (23.5)	1344 (24.7)	318 (19.4)	**<0.001**	667 (21.7)	560 (23.4)	107 (15.6)	**<0.001**	995 (24.8)	784 (25.7)	211 (22.0)	**0.022**
Mineralocorticoid receptor antagonists	573 (8.1)	467 (8.6)	106 (6.5)	**0.005**	265 (8.6)	226 (9.5)	39 (5.7)	**0.002**	308 (7.7)	241 (7.9)	67 (7.0)	0.359
Calcium channel blockers	2047 (28.9)	1621 (29.8)	426 (25.9)	**0.002**	815 (26.5)	662 (27.7)	153 (22.3)	**0.005**	1232 (30.8)	959 (31.5)	273 (28.5)	0.086
Statins	4721 (66.7)	3528 (64.9)	1193 (72.7)	**<0.001**	2077 (67.5)	1580 (66.1)	497 (72.6)	**0.001**	2644 (66.0)	1948 (63.9)	696 (72.7)	**<0.001**
Lipid-lowering agents other than statins ^d^	589 (8.3)	438 (8.1)	151 (9.2)	0.142	244 (7.9)	184 (7.7)	60 (8.8)	0.365	345 (8.6)	254 (8.3)	91 (9.5)	0.258

In the case of categorical variables, values representing the frequency and the percentages are provided in parentheses (% of n). In the case of continuous variables, means ± standard deviations are provided. CV—cardiovascular; TIA—transient ischaemic attack; MI—myocardial infarction; COPD—chronic obstructive pulmonary disease; ACEI—angiotensin-converting enzyme inhibitor; ARB—angiotensin receptor blocker; *p*—statistical significance between persistent and non-persistent patients according to the χ^2^-test; * statistical significance according to the Mann–Whitney U test; in the case of statistical significance (*p* < 0.05), the values are expressed in bold. ^a^ The time period covered by “history”—5 years before the index date of this study. ^b^ New user of ACEIs/ARBs—patient in whom ACEI/ARB treatment was initiated in association with the diagnosis of peripheral arterial disease. ^c^ Co-payment—calculated as the cost of ACEI/ARB treatment paid by the patient per month. ^d^ Lipid-lowering agents other than statins–ezetimibe and fibrates.

**Table 2:** Multivariate analysis of the association between patient- and medication-related characteristics, and the probability of non-persistence in the whole study cohort and in the groups of males and females.

Factor	The Whole Study Cohort (*n* = 7080)	Males (*n* = 3075)	Females (*n* = 4005)
*Socio-demographic characteristics*			
Age	0.99 (0.98–1.00)	1.00 (0.98–1.01)	**0.98 (0.97–0.99)**
Female sex	1.08 (0.97–1.21)		
University education	0.98 (0.80–1.19)	1.04 (0.82–1.31)	0.79 (0.53–1.19)
Employed patients	0.87 (0.69–1.10)	0.77 (0.57–1.05)	1.06 (0.73–1.53)
*History of CV events* ^a^			
History of ischemic stroke	0.98 (0.85–1.13)	0.90 (0.72–1.13)	1.03 (0.85–1.25)
History of TIA	1.06 (0.87–1.30)	1.14 (0.82–1.60)	1.00 (0.78–1.29)
History of MI	0.97 (0.77–1.22)	1.01 (0.71–1.42)	0.94 (0.69–1.28)
*Comorbid conditions*			
Number of comorbid conditions	1.03 (0.93–1.14)	0.92 (0.79–1.07)	1.11 (0.98–1.27)
Chronic heart failure	1.04 (0.82–1.32)	1.36 (0.93–2.00)	0.91 (0.67–1.23)
Atrial fibrillation	**0.81 (0.66–0.99)**	0.98 (0.72–1.34)	**0.68 (0.51–0.89)**
Diabetes mellitus	**0.82 (0.71–0.95)**	0.87 (0.69–1.09)	**0.78 (0.64–0.95)**
Hypercholesterolemia	0.95 (0.82–1.10)	0.92 (0.73–1.16)	0.95 (0.78–1.16)
Dementia	**0.78 (0.62–0.99)**	0.92 (0.61–1.38)	**0.73 (0.54–0.98)**
Depression	1.10 (0.91–1.32)	1.13 (0.80–1.61)	1.02 (0.81–1.28)
Anxiety disorders	1.02 (0.88–1.19)	1.16 (0.89–1.50)	0.95 (0.78–1.15)
Parkinson’s disease	1.02 (0.77–1.34)	1.10 (0.67–1.80)	0.94 (0.67–1.32)
Epilepsy	1.04 (0.76–1.44)	1.23 (0.76–1.98)	0.91 (0.58–1.41)
Bronchial asthma/COPD	1.04 (0.89–1.23)	1.04 (0.80–1.34)	1.03 (0.83–1.28)
*ACEI/ARB related characteristics*			
Initially administered ACEI/ARB			
Perindopril	1.00	1.00	1.00
Lisinopril	1.10 (0.88–1.37)	1.09 (0.77–1.55)	1.11 (0.83–1.48)
Ramipril	0.88 (0.75–1.03)	**0.74 (0.59–0.94)**	1.01 (0.82–1.24)
Enalapril	**0.51 (0.29–0.89)**	0.51 (0.23–1.16)	0.50 (0.24–1.07)
Spirapril	1.19 (0.38–3.70)	0.67 (0.09–4.83)	1.76 (0.43–7.16)
Trandolapril	0.88 (0.75–1.02)	**0.79 (0.62–0.99)**	0.94 (0.77–1.15)
Quinapril	0.84 (0.69–1.03)	**0.67 (0.47–0.96)**	0.95 (0.74–1.21)
Imidapril	**1.68 (1.18–2.39)**	**2.11 (1.20–3.73)**	1.53 (0.97–2.41)
Fosinopril	**1.56 (1.02–2.40)**	1.37 (0.64–2.93)	1.68 (0.99–2.83)
Valsartan	**1.65 (1.25–2.19)**	1.30 (0.82–2.06)	**1.98 (1.39–2.82)**
Losartan	0.71 (0.47–1.08)	0.53 (0.26–1.08)	0.88 (0.53–1.47)
Telmisartan	1.02 (0.69–1.50)	0.81 (0.42–1.59)	1.14 (0.71–1.84)
Candesartan	0.90 (0.58–1.40)	0.65 (0.27–1.58)	1.08 (0.65–1.80)
Irbesartan	1.72 (0.64–4.61)	1.54 (0.38–6.26)	1.89 (0.47–7.71)
New user of ACEIs/ARBs ^b^	**2.05 (1.70–2.47)**	**1.53 (1.11–2.11)**	**2.50 (1.98–3.15)**
Patient’s co-payment (EUR) ^c^	0.98 (0.97–1.01)	0.98 (0.95–1.01)	0.99 (0.97–1.01)
General practitioner as index prescriber	**0.70 (0.62–0.79)**	**0.70 (0.58–0.84)**	**0.70 (0.60–0.82)**
*CV co-medication*			
Number of medications	**0.95 (0.93–0.98)**	**0.96 (0.92–0.99)**	**0.95 (0.92–0.98)**
Number of CV medications	**1.08 (1.03–1.14)**	1.06 (0.97–1.15)	**1.09 (1.02–1.16)**
Antiplatelet agents	0.93 (0.83–1.05)	0.97 (0.79–1.18)	0.91 (0.78–1.07)
Anticoagulants	0.93 (0.80–1.07)	1.00 (0.80–1.26)	0.89 (0.74–1.07)
Cardiac glycosides	0.84 (0.67–1.05)	0.79 (0.53–1.16)	0.89 (0.68–1.18)
Antiarrhythmic agents	0.96 (0.77–1.21)	0.96 (0.68–1.36)	1.00 (0.74–1.35)
Beta-blockers	**0.86 (0.74–0.99)**	**0.77 (0.59–0.99)**	0.92 (0.77–1.11)
Thiazide diuretics	**0.78 (0.68–0.90)**	0.81 (0.64–1.02)	**0.77 (0.64–0.92)**
Loop diuretics	0.94 (0.80–1.11)	0.87 (0.66–1.15)	0.98 (0.81–1.20)
Mineralocorticoid receptor antagonists	1.04 (0.82–1.31)	0.88 (0.60–1.30)	1.21 (0.90–1.62)
Calcium channel blockers	**0.82 (0.71–0.93)**	0.82 (0.66–1.02)	**0.83 (0.70–0.98)**
Statins	**1.18 (1.04–1.34)**	1.20 (0.98–1.46)	**1.20 (1.02–1.41)**
Lipid-lowering agents other than statins ^d^	1.09 (0.91–1.30)	1.13 (0.84–1.51)	1.06 (0.84–1.33)

Values represent hazard ratios (95% confidence intervals). In the case of statistical significance (*p* < 0.05), the values are expressed in bold. CV—cardiovascular; TIA—transient ischaemic attack; MI—myocardial infarction; COPD—chronic obstructive pulmonary disease; ACEI—angiotensin-converting enzyme inhibitor; ARB—angiotensin receptor blocker. ^a^ The time period covered by “history”—5 years before the index date of this study. ^b^ New user of ACEIs/ARBs—patient in whom ACEI/ARB treatment was initiated in association with the diagnosis of peripheral arterial disease. ^c^ Co-payment—calculated as the cost of ACEI/ARB treatment paid by the patient per month. ^d^ Lipid lowering agents other than statins—ezetimibe and fibrates.

## Data Availability

The data that support the findings of this study are available from the General Health Insurance Company, but restrictions apply to the availability of these data, which were used under license for the current study, and so are not publicly available. Data are, however, available from the authors upon reasonable request and with the permission of the General Health Insurance Company.

## Supplementary Materials

The following are available online at https://www.mdpi.com/article/10.3390/biomedicines10071479/s1: Supplementary Table S1. Sensitivity analysis of the effect of different lengths of treatment gap period defining non-persistence on the proportion of non-persistent patients; Supplementary Table S2. Multivariate analysis of the association between patient- and medication-related characteristics and the probability of non-persistence in the models with a 3-year follow-up period.
