# Supplementary material for: Gender Differences in Non-Persistence with Angiotensin-Converting Enzyme Inhibitors and Angiotensin Receptor Blockers among Older Hypertensive Patients with Peripheral Arterial Disease
Source: Biomedicines. 2022 Jun 22;10(7):1479. doi: 10.3390/biomedicines10071479 (PMC9313155; doi:10.3390/biomedicines10071479)
Supplement: Supplementary file 1 [file biomedicines-10-01479-s001.zip › Supplementary Table S1.pdf]

Supplementary Table S1. Sensitivity analysis of the effect of different lengths of treatment gap period defining non-persistence on the proportion of non-persistent patients.

	Treatment gap to define non-persistence (months)						
	1	2	3	4	5	6	12
The whole study cohort (n = 7080)							
1st year	2224 (31.4)	1395 (19.7)	1034 (14.6)	820 (11.6)	721 (10.2)	664 (9.4)	488 (6.9)
2nd year	646 (9.1)	579 (8.2)	498 (7.0)	455 (6.4)	400 (5.7)	365 (5.2)	313 (4.4)
3rd year	309 (4.4)	342 (4.8)	323 (4.6)	300 (4.2)	283 (4.0)	272 (3.8)	239 (3.4)
4th year	192 (2.7)	236 (3.3)	241 (3.4)	245 (3.5)	243 (3.4)	220 (3.1)	194 (2.7)
5th year	141 (2.0)	180 (2.6)	174 (2.5)	149 (2.1)	134 (1.9)	121 (1.7)	15 (0.2)
Total	3512 (49.6)	2732 (38.6)	2270 (32.1)	1969 (27.8)	1781 (25.2)	1642 (23.2)	1249 (17.6)
Males (n = 3075)							
1st year	935 (30.4)	574 (18.7)	420 (13.6)	338 (11.0)	300 (9.8)	274 (8.9)	197 (6.4)
2nd year	287 (9.3)	231 (7.5)	200 (6.5)	183 (6.0)	160 (5.2)	143 (4.6)	124 (4.0)
3rd year	127 (4.1)	145 (4.7)	131 (4.3)	126 (4.1)	124 (4.0)	119 (3.9)	104 (3.4)
4th year	81 (2.7)	103 (3.4)	114 (3.7)	111 (3.6)	108 (3.5)	98 (3.2)	87 (2.8)
5th year	55 (1.8)	78 (2.5)	76 (2.5)	67 (2.1)	60 (2.0)	51 (1.7)	6 (0.2)
Total	1485 (48.3)	1131 (36.8)	941 (30.6)	825 (26.8)	752 (24.5)	685 (22.3)	518 (16.8)
Females (n = 4005)							
1st year	1289 (32.2)	821 (20.5)	614 (15.3)	482 (12.0)	421 (10.5)	390 (9.7)	291 (7.3)
2nd year	359 (9.0)	348 (8.7)	298 (7.4)	272 (6.8)	240 (6.0)	222 (5.5)	189 (4.7)
3rd year	182 (4.5)	197 (4.9)	192 (4.8)	174 (4.3)	159 (4.0)	153 (3.8)	135 (3.4)
4th year	111 (2.8)	133 (3.3)	127 (3.2)	134 (3.4)	135 (3.4)	122 (3.1)	107 (2.7)
5th year	86 (2.1)	102 (2.6)	98 (2.5)	82 (2.1)	74 (1.8)	70 (1.8)	9 (0.2)
Total	2027 (50.6)	1601 (40.0)	1329 (33.2)	1144 (28.6)	1029 (25.7)	957 (23.9)	731 (18.3)

Values represent the frequency of non-persistent patients, the percentages are provided in parentheses (% of n).
