# Supplementary material for: Gender Differences in Non-Persistence with Angiotensin-Converting Enzyme Inhibitors and Angiotensin Receptor Blockers among Older Hypertensive Patients with Peripheral Arterial Disease
Source: Biomedicines. 2022 Jun 22;10(7):1479. doi: 10.3390/biomedicines10071479 (PMC9313155; doi:10.3390/biomedicines10071479)
Supplement: Supplementary file 1 [file biomedicines-10-01479-s001.zip › Supplementary Table S2.pdf]

Supplementary Table S2. Multivariate analysis of the association between patient- and medication-related characteristics and the probability of non-persistence in the models with a 3-year follow-up period.

Factor	The whole study cohort (n = 7080)	Males (n = 3075)	Females (n = 4005)
Socio-demographic characteristics			
Age	1.00 (0.99–1.01)	0.99 (0.98–1.01)	1.00 (0.99–1.02)
Female sex	1.09 (0.97–1.22)		
University education	0.91 (0.73–1.14)	0.97 (0.75–1.25)	0.74 (0.46–1.18)
Employed patients	0.94 (0.73–1.21)	0.87 (0.63–1.20)	1.04 (0.69–1.58)
History of CV events[a]			
History of ischemic stroke	1.09 (0.94–1.26)	0.98 (0.78–1.23)	1.19 (0.99–1.44)
History of TIA	1.05 (0.85–1.30)	1.07 (0.74–1.56)	1.02 (0.78–1.33)
History of MI	1.01 (0.80–1.28)	1.17 (0.83–1.64)	0.89 (0.64–1.23)
Comorbid conditions			
Number of comorbid conditions	1.02 (0.92–1.13)	0.97 (0.83–1.13)	1.04 (0.90–1.20)
Chronic heart failure	1.08 (0.85–1.37)	**1.50 (1.03–2.18)**	0.92 (0.67–1.26)
Atrial fibrillation	0.83 (0.67–1.03)	0.97 (0.71–1.34)	**0.72 (0.54–0.97)**
Diabetes mellitus	0.86 (0.74–1.01)	**0.78 (0.61–0.98)**	0.93 (0.75–1.14)
Hypercholesterolemia	1.00 (0.86–1.17)	0.91 (0.71–1.16)	1.07 (0.87–1.32)
Dementia	0.91 (0.72–1.15)	1.07 (0.72–1.58)	0.86 (0.64–1.15)
Depression	1.09 (0.89–1.32)	0.93 (0.63–1.37)	1.09 (0.86–1.39)
Anxiety disorders	0.96 (0.81–1.13)	0.98 (0.74–1.29)	0.95 (0.77–1.17)
Parkinson's disease	0.99 (0.74–1.32)	1.18 (0.73–1.92)	0.93 (0.65–1.33)
Epilepsy	1.03 (0.74–1.45)	1.21 (0.74–1.97)	0.90 (0.56–1.44)
Bronchial asthma/COPD	1.07 (0.90–1.28)	1.02 (0.78–1.33)	1.11 (0.88–1.40)
ACEI/ARB-related characteristics			
Initially administered ACEI/ARB			
Perindopril	1.00	1.00	1.00
Lisinopril	0.96 (0.75–1.23)	0.98 (0.67–1.44)	0.95 (0.69–1.31)
Ramipril	0.92 (0.78–1.08)	**0.76 (0.59–0.97)**	1.07 (0.86–1.33)
Enalapril	**0.52 (0.28–0.94)**	0.54 (0.22–1.31)	0.51 (0.22–1.15)
Spirapril	1.31 (0.42–4.09)	0.84 (0.12–6.05)	1.90 (0.47–7.72)
Trandolapril	0.89 (0.75–1.04)	0.81 (0.63–1.03)	0.95 (0.77–1.18)
Quinapril	0.83 (0.67–1.03)	0.69 (0.47–1.02)	0.92 (0.70–1.20)
Imidapril	**1.68 (1.16–2.43)**	1.84 (0.99–3.41)	**1.63 (1.02–2.61)**
Fosinopril	0.86 (0.47–1.57)	0.89 (0.33–2.40)	0.86 (0.40–1.82)
Valsartan	**1.65 (1.24–2.20)**	**1.57 (1.01–2.44)**	**1.72 (1.17–2.52)**
Losartan	0.70 (0.44–1.09)	0.84 (0.45–1.56)	0.57 (0.29–1.11)
Telmisartan	0.90 (0.58–1.41)	0.75 (0.35–1.61)	0.98 (0.56–1.72)
Candesartan	0.85 (0.51–1.40)	0.48 (0.15–1.51)	1.07 (0.61–1.88)
Irbesartan	2.02 (0.83–4.91)	1.38 (0.34–5.65)	2.58 (0.81–8.22)
New user of ACEI/ARB[b]	**2.17 (1.80–2.62)**	**1.90 (1.40–2.58)**	**2.46 (1.94–3.13)**
Patient´s co-payment (EUR)[c]	0.99 (0.97–1.01)	0.98 (0.96–1.01)	1.00 (0.97–1.02)
General practitioner as index prescriber	**0.69 (0.61–0.79)**	**0.74 (0.60–0.90)**	**0.66 (0.56–0.78)**

(Table continued)

Factor	The whole study cohort (n = 7080)	Males (n = 3075)	Females (n = 4005)
CV co-medication			
Number of medications	**0.93 (0.90–0.95)**	**0.93 (0.90–0.97)**	**0.92 (0.89–0.96)**
Number of CV medications	**1.13 (1.07–1.19)**	**1.10 (1.01–1.20)**	**1.14 (1.07–1.22)**
Antiplatelet agents	0.93 (0.82–1.06)	0.94 (0.76–1.15)	0.93 (0.79–1.10)
Anticoagulants	1.00 (0.86–1.15)	1.05 (0.83–1.33)	0.97 (0.80–1.17)
Cardiac glycosides	**0.79 (0.63–0.99)**	0.87 (0.60–1.25)	0.77 (0.58–1.02)
Antiarrhythmic agents	1.00 (0.79–1.25)	1.01 (0.72–1.43)	1.03 (0.76–1.39)
Beta-blockers	**0.77 (0.66–0.90)**	**0.67 (0.51–0.89)**	0.83 (0.68–1.01)
Thiazide diuretics	**0.80 (0.69–0.93)**	**0.71 (0.55–0.92)**	0.84 (0.70–1.02)
Loop diuretics	0.97 (0.82–1.14)	0.95 (0.72–1.26)	0.96 (0.78–1.18)
Mineralocorticoid receptor antagonists	0.95 (0.75–1.20)	0.71 (0.48–1.05)	1.18 (0.88–1.59)
Calcium channel blockers	**0.78 (0.68–0.90)**	**0.73 (0.57–0.92)**	**0.82 (0.69–0.98)**
Statins	1.06 (0.93–1.21)	1.03 (0.84–1.26)	1.10 (0.92–1.30)
Lipid lowering agents other than statins[d]	1.10 (0.91–1.33)	1.07 (0.78–1.46)	1.10 (0.86–1.41)

Values represent hazard ratios (95% confidence intervals). In the case of statistical significance ($p<0.05$), the values are expressed in bold. CV – cardiovascular; TIA – transient ischemic attack; MI – myocardial infarction; COPD – chronic obstructive pulmonary disease; ACEI – angiotensin-converting enzyme inhibitor; ARB – angiotensin receptor blocker. [a] The time period covered by "history" – 5 years before the index date of this study. [b] New user of ACEIs/ARBs – patient in whom ACEI/ARB treatment was initiated in association with the diagnosis of peripheral arterial disease. [c] Co-payment – calculated as the cost of ACEI/ARB treatment paid by the patient per month. [d] Lipid lowering agents other than statins – ezetimibe and fibrates.
